# Immunohistochemical analysis of MMP-1 and 2 in patients with lichen planopilaris and frontal fibrosing alopecia^؆^

**DOI:** 10.1016/j.abd.2026.501359

**Published:** 2026-05-19

**Authors:** Renata Indelicato Zac, Emily Ferreira Salles Pilar, Julio Cesar Moraes, Isabel Cristina Gomes Moura, Moisés Salgado Pedrosa, Adilson da Costa

**Affiliations:** a Department of Dermatology, Clínica Renata Zac, Belo Horizonte, MG, Brazil; b Department of Biology, Hospital das Clínicas de Porto Alegre, Porto Alegre, RS, Brazil; c Processing Department, Imunocell Laboratorial, Campinas, SP, Brazil; d Department of Statistics, Faculdade de Ciências Médicas de Minas Gerais, Belo Horizonte, MG, Brazil; e Department of Pathology, Centro Especializado em Anatomia Patológica, Belo Horizonte, MG, Brazil; f Instituto de Assistencia Medica ao Servidor Publico Estadual, Sao Paulo, SP, Brazil; Emory University Department of Dermatology, Atlanta, GA, USA

Dear Editor,

Lichen planopilaris (LPP) and frontal fibrosing alopecia (FFA) are primary lymphocytic scarring alopecias. Clinically, classic LPP shows an area of irregular alopecia that is more common in the vertex region, while FFA causes hair loss that occurs slowly in the frontotemporal implantation hairline, commonly associated with eyebrow alopecia.^1^

According to Doche et al.,^2^ approximately 65% of the scalp biopsy specimens from “normal-appearing” areas of LPP and FFA showed perifollicular inflammation around the isthmus/infundibulum region. These findings suggest that both diseases may be more generalized processes affecting the scalp. The lack of relationship between the degree of histopathological inflammation and clinical signs of inflammation supports the idea that some scalp areas could possibly be more prone to develop a cicatricial alopecia.

These disorders are mediated by lymphocytes and result in disruption of the basement membrane cells, destruction of the hair follicle and replacement by fibrosis. They have a chronic course, unpredictable development and, probably, autoimmune pathogenesis. Drugs, infections, genetic factors and immunological abnormalities are possible triggering factors.^3^

Matrix metalloproteinases (MMPs) are involved in cell proliferation and differentiation, inflammation, degeneration, tumor metastasis, and growth. The production and activation of MMPs is rapidly induced when active tissue remodeling is needed. MMP-2, the most widely distributed of all MMPs, along with MMP-9, is said to digest type IV, V and XI collagens; laminin and aggrecan core protein, and singly digests collagens I, II and III.^4^

MMP-1 is capable of degrading type I and III fibrillar collagens.^5^

MMP-4 (or MMP-17) is involved in different pathological processes such as arthritis, cardiovascular disease, and cancer progression.^6^

Our objective was to evaluate the presence of MMP-1 and MMP-2 in LPP and FFA and to assess their role in the pathogenesis of these diseases.

The patients were collected from Renata Zac Dermatological Clinic, Belo Horizonte, MG, Brazil, from 2019 to 2023.

A university ethical committee approval was obtained prior to carrying out this study. Participants taking part gave written informed consent.

Inclusion criteria were patients with clinically and histopathologically proven disease. Patients with lesions not confirmed by biopsy or on treatment at the time of biopsy were excluded from this study.

In this study, 60 patients (48 females and 12 males) aged from 23 to 71 years were included.

To be included in the classical LPP group, the patient should have an irregular involvement of the scalp in the form of plaques presenting with hyperkeratosis, follicular plugs and perifollicular erythema or atrophic scars, mainly in the apex and in the parietal region. Trichoscopy could evidence perifollicular and interfollicular erythema associated with tubular perifollicular scales. Complaints of pruritus and burning sensation were common, as well as the association with the skin, nail and mucosal LP lesions.^3^

To be included in the FFA group, the participants should fulfill the diagnostic criteria for FFA proposed by Vañó-Galván et al.^7^ The major criteria are scarring alopecia of the scalp in the frontotemporal region (in the absence of keratotic follicular papules on the body) and bilateral diffuse alopecia of the eyebrows. The minor criteria include trichoscopy with peripilar erythema, peripilar desquamation, or both; histopathological characteristics of scarring alopecia with FFA or LPP pattern; involvement of occipital region, face, sideburns, body hair and presence of non-inflammatory facial papules. The diagnosis requires two major criteria or one major and two minor criteria.

All patients had undergone a scalp biopsy with a 4-mm punch biopsy, with one sample submitted for vertical sections and the other for cross-sectional sections. Diagnosis was confirmed by one pathologist and one dermatologist using hematoxylin and eosin-stained sections. The histopathological alterations found in LPP and FFA are a perifollicular lymphohistiocytic infiltrate, sometimes with a lichenoid pattern, more prominent in the isthmus and infundibulum regions; vacuolar degeneration of basal cells; necrotic keratinocytes; artifactual clefts between the follicle and the perifollicular fibrous band; and perifollicular fibrosis separating the inflammatory infiltrate from the follicle. Over time, there is a reduction and loss of sebaceous glands and destruction of the entire hair follicle.^1^

Twenty apparently normal scalp tissues, age and sex matched, were obtained at the time of facial plastic surgery and used as controls.

This laboratory-based study involved the use of sixty buffered, formalin-fixed, paraffin-embedded tissue blocks of histologically proven cases of LLP, FFA and controls.

The primary antibodies for immunohistochemistry staining included polyclonal antibodies against MMP1 [EP1247Y] (ab52631, Abcam, Cambridge, UK) AND MMP2 [6E3F8] (ab86607, Abcam, Cambridge, UK).

The expression of MMP-1 and MMP-2 in LPP, FFA, and control groups was evaluated in the sebaceous glands (SG), all segments of the hair follicles (HF), and the epidermal keratinocytes (KT).

The Klein score is a semiquantitative method used to evaluate the expression of immunohistochemical markers by multiplying the percentage of positive cells by the staining intensity to obtain the final score (Klein et al., 2001).

The Klein score was obtained through the calculation described in Table 1: Calculation of the Klein score.

**Table 1:** Calculation of the Klein score.

% of positive cells (A)	Intensity (B)	Final score
0 = 0%	0 = No reaction	A × B
1 = 1% - 29%	1 = Weak reaction	
2 = 30% - 60%	2 = Moderate reaction	
3 = 61% - 100%	3 = Strong reaction	

Qualitative variables were presented as absolute and relative frequencies, and quantitative variables as minimum, maximum, mean, standard deviation, median, first (Q1) and third (Q3) quartiles. The number of available information (valid n) was presented for each variable.

The association between qualitative variables was assessed using Fisher's exact test. The comparison of quantitative variables between the three comparison groups was performed using the Kruskal-Wallis test.

The analyses were carried out in the R Studio program version 2024.04.2 using the *R* language version 4.4.0, and p < 0.05 was considered significant.

There was no difference between the three groups evaluated in the MMP-1 and MMP-2 antibody markers (Figure 1, Figure 2 and Table 2, Table 3).

**Figure 1:**
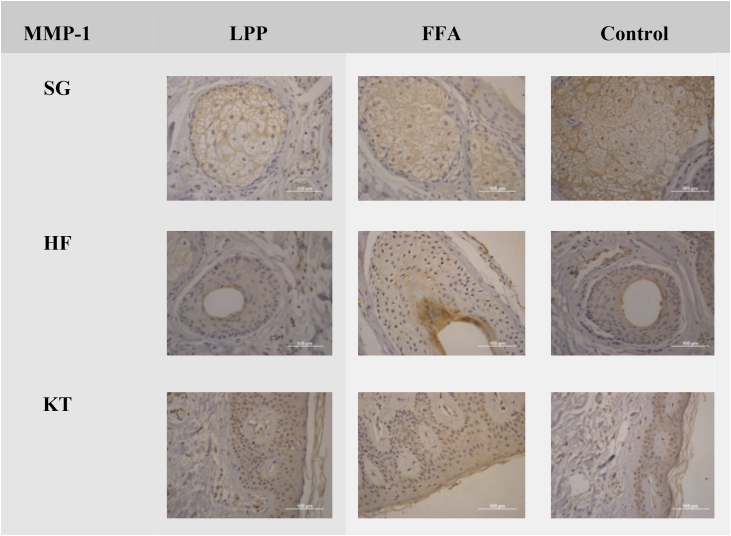
Negative expression of MMP-1 in LPP, FFA, and control groups in the sebaceous glands (SG), hair follicles (HF) and keratinocytes (KT) ×100. Source: research original data.

**Figure 2:**
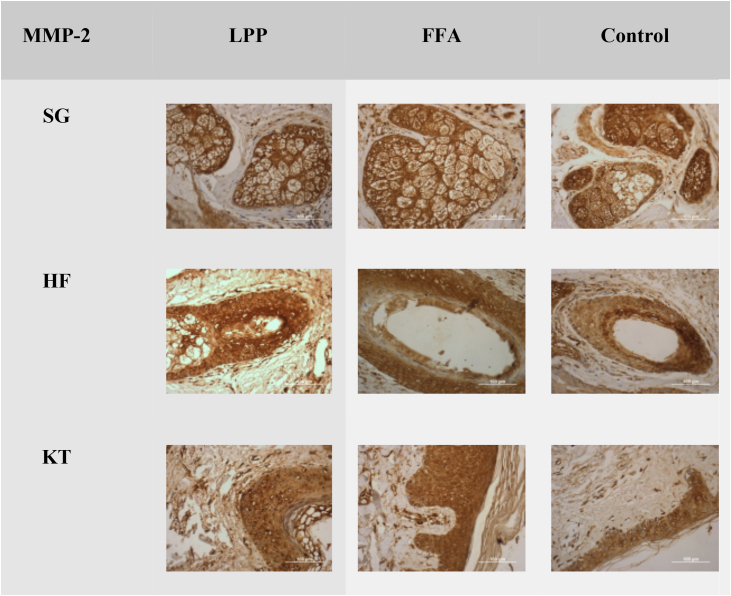
Negative expression of MMP-2 in LPP, FFA, and control groups in the sebaceous glands (SG), hair follicles (HF) and keratinocytes (KT) ×100. Source: research original data.

**Table 2:** MMP1 antibody data according to the groups evaluated.

Variables	Control	FFA	LPP	p-value
***Sebaceous glands***				
% Positive cells				‒
Min/Max	100.0/100.0	100.0/100.0	100.0/100.0	
Median [Q1; Q3]	100.0 [100.0; 100.0]	100.0 [100.0; 100.0]	100.0 [100.0; 100.0]	
Standard deviation	100.0 (0)	100.0 (0)	100.0 (0)	
N	19	15	15	
Dominant intensity				0.057^a^
No reaction	0 (‒)	0 (‒)	0 (‒)	
Weak reaction	3 (15.8%)	7 (46.7%)	9 (60.0%)	
Moderate reaction	14 (73.7%)	6 (40.0%)	5 (33.3%)	
Strong reaction	2 (10.5%)	2 (13.3%)	1 (6.7%)	
N	19	15	15	
Place				
Bulb	5 (26.3%)	5 (33.3%)	1 (6.7%)	0.207^a^
Loney hair	0 (-)	1 (6.7%)	2 (13.3%)	0.273^a^
Transversal hair	9 (47.4%)	5 (33.3%)	5 (33.3%)	0.617^a^
Upper portion	6 (31.6%)	3 (20.0%)	7 (46.7%)	0.326^a^
N	19	15	15	
Klein score				0.061^b^
Min/Max	3.0/9.0	3.0/9.0	3.0/9.0	
Median [Q1; Q3]	6.0 [6.0; 6.0]	6.0 [3.0; 6.0]	3.0 [3.0; 6.0]	
Standard deviation	5.8 (1.6)	5.0 (2.2)	4.4 (1.9)	
N	19	15	15	
***Hair follicle***				
% Positive cells				0.922^b^
Min/Max	0/100.0	0/100.0	0/100.0	
Median [Q1; Q3]	75.0 [57.5; 90.0]	70.0 [30.0; 100.0]	80.0 [20.0; 97.5]	
Standard deviation	69.5 (27.8)	61.1 (39.6)	58.6 (41.9)	
N	20	19	18	
Dominant intensity				0.551^a^
No reaction	1 (5.0%)	4 (21.1%)	4 (22.2%)	
Weak reaction	15 (75.0%)	12 (63.2%)	10 (55.6%)	
Moderate reaction	4 (20.0%)	3 (15.8%)	3 (16.7%)	
Strong reaction	0 (-)	0 (-)	1 (5.6%)	
N	20	19	18	
Klein score				0.620^b^
Min/Max	0/6.0	0/6.0	0/9.0	
Median [Q1; Q3]	3.0 [2.0; 3.0]	3.0 [2.0; 3.0]	3.0 [1.0; 3.0]	
Standard deviation	3.1 (1.6)	2.6 (1.9)	2.8 (2.4)	
N	20	19	18	
***Keratinocyte***				
% Positive cells				0.780^b^
Min/Max	0/100.0	0/100.0	0/100.0	
Mediana [Q1; Q3]	100.0 [25.0; 100.0]	92.5 [7.5; 100.0]	90.0 [20.0; 100.0]	
Média (dp)	71.1 (40.0)	63.2 (44.7)	62.6 (41.1)	
N válido	19	20	19	
Dominant intensity				0.505^a^
No reaction	2 (10.5%)	5 (25.0%)	1 (5.3%)	
Weak reaction	13 (68.4%)	10 (50.0%)	13 (68.4%)	
Moderate reaction	4 (21.1%)	5 (25.0%)	4 (21.1%)	
Strong reaction	0 (-)	0 (-)	1 (5.3%)	
N	19	20	19	
Klein score				0.971^b^
Min/Max	0/6.0	0/6.0	0/9.0	
Median [Q1; Q3]	3.0 [1.5; 3.0]	3.0 [0.8; 3.8]	3.0 [1.0; 4.5]	
Standard deviation	2.9 (1.9)	2.8 (2.2)	3.1 (2.4)	
N	19	20	19	
Layer				0.100^a^
Basal	0 (‒)	0 (‒)	3 (16.7%)	
Basal and Parabasal	17 (100.0%)	15 (100.0%)	15 (83.3%)	
N	17	15	18	
***Limphocytes***				
Presence				‒
Negative	0 (‒)	0 (‒)	0 (‒)	
Positive	14 (100.0%)	19 (100.0%)	15 (100.0%)	
N válido	14	19	15	
Dominant intensity				0.731^a^
No reaction	5 (35.7%)	3 (15.8%)	2 (13.3%)	
Weak reaction	4 (28.6%)	10 (52.6%)	8 (53.3%)	
Moderate reaction	4 (28.6%)	4 (21.1%)	4 (26.7%)	
Strong reaction	1 (7.1%)	2 (10.5%)	1 (6.7%)	
N	14	19	15	

a Teste Exato de Fisher.

b Teste de Kruskal-Wallis.

**Table 3:** MMP2 antibody data according to the groups evaluated.

Variables	Control	FFA	LPP	p-value
***Sebaceous glands***				
% Positive cells				‒
Min/Max	100.0/100.0	100.0/100.0	100.0/100.0	
Median [Q1; Q3]	100.0 [100.0; 100.0]	100.0 [100.0; 100.0]	100.0 [100.0; 100.0]	
Standard deviation	100.0 (0)	100.0 (0)	100.0 (0)	
N	19	13	16	
Dominant intensity				0.602^a^
No reaction	0 (‒)	0 (‒)	0 (‒)	
Weak reaction	0 (‒)	0 (‒)	0 (‒)	
Moderate reaction	1 (5.3%)	2 (15.4%)	2 (12.5%)	
Strong reaction	18 (94.7%)	11 (84.6%)	14 (87.5%)	
N	19	13	16	
Place				
Bulb	3 (15.8%)	5 (38.5%)	1 (6.2%)	0.103^a^
Loney hair	0 (‒)	0 (‒)	2 (12.5%)	0.176^a^
Transversal hair	10 (52.6%)	7 (53.8%)	4 (25.0%)	0.203^a^
Upper portion	8 (42.1%)	3 (23.1%)	8 (50.0%)	0.323^a^
N	19	13	16	
Klein score				0.625^b^
Min/Max	6.0/9.0	6.0/9.0	6.0/9.0	
Median [Q1; Q3]	9.0 [9.0; 9.0]	9.0 [9.0; 9.0]	9.0 [9.0; 9.0]	
Standard deviation	8.8 (0.7)	8.5 (1.1)	8.6 (1.0)	
N	19	13	16	
***Hair follicle***				
% Positive cells				0.360^b^
Min/Max	100.0/100.0	70.0/100.0	90.0/100.0	
Median [Q1; Q3]	100.0 [100.0; 100.0]	100.0 [100.0; 100.0]	100.0 [100.0; 100.0]	
Standard deviation	100.0 (0)	98.0 (7.0)	99.4 (2.4)	
N	20	20	18	
Dominant intensity				0.883^a^
No reaction	0 (‒)	0 (‒)	0 (‒)	
Weak reaction	2 (10.0%)	3 (15.0%)	3 (16.7%)	
Moderate reaction	12 (60.0%)	9 (45.0%)	9 (50.0%)	
Strong reaction	6 (30.0%)	8 (40.0%)	6 (33.3%)	
N	20	20	18	
Klein score				0.914^b^
Min/Max	3.0/9.0	3.0/9.0	3.0/9.0	
Median [Q1; Q3]	6.0 [6.0; 9.0]	6.0 [6.0; 9.0]	6.0 [6.0; 9.0]	
Standard deviation	6.6 (1.8)	6.8 (2.1)	6.5 (2.1)	
N	20	20	18	
***Keratinocyte***				
% Positive cells				0.387^b^
Min/Max	100.0/100.0	100.0/100.0	10.0/100.0	
Mediana [Q1; Q3]	100.0 [100.0; 100.0]	100.0 [100.0; 100.0]	100.0 [100.0; 100.0]	
Média (dp)	100.0 (0)	100.0 (0)	95.5 (20.1)	
N válido	18	20	20	
Dominant intensity				0.933^a^
No reaction	0 (‒)	0 (‒)	0 (‒)	
Weak reaction	3 (16.7%)	4 (20.0%)	5 (25.0%)	
Moderate reaction	9 (50.0%)	10 (50.0%)	11 (55.0%)	
Strong reaction	6 (33.3%)	6 (30.0%)	4 (20.0%)	
N	18	20	20	
Klein score				0.575^b^
Min/Max	3.0/9.0	3.0/9.0	1.0/9.0	
Median [Q1; Q3]	6.0 [6.0; 9.0]	6.0 [6.0; 9.0]	6.0 [5.2; 6.0]	
Standard deviation	6.5 (2.1)	6.3 (2.2)	5.8 (2.2)	
N	18	20	20	
Layer				‒
Basal	0 (‒)	0 (‒)	0 (‒)	
Basal and Parabasal	18 (100.0%)	20 (100.0%)	20 (100.0%)	
N	18	20	20	
***Limphocytes***				
Presence				‒
Negative	0 (‒)	0 (‒)	0 (‒)	
Positive	13 (100.0%)	19 (100.0%)	13 (100.0%)	
N válido	13	19	13	
Dominant intensity				0.086^a^
No reaction	0 (‒)	0 (‒)	0 (‒)	
Weak reaction	0 (‒)	1 (5.3%)	3 (23.1%)	
Moderate reaction	1 (7.7%)	3 (15.8%)	4 (30.8%)	
Strong reaction	12 (92.3%)	15 (78.9%)	6 (46.2%)	
N	13	19	13	

a Teste Exato de Fisher.

b Teste de Kruskal-Wallis.

LPP and FFA are primary lymphocytic scarring alopecias in which the lower infundibular and isthmic areas of the hair follicle are primarily affected, the region where follicular stem cells are located.^2^

It has been observed that many patients being followed for LPP and FFA, without inflammatory symptoms or signs, continue with the progression of the area of alopecia.^1^

Wong and Goldberg,^8^ found statistically significant differences between the extent of the inflammatory infiltrate below the isthmus in FFA when compared with LPP (92% vs. 63%; p = 0.02) but found no differences between the inflammatory infiltrate intensity in the two diseases.

Although the pathophysiology of both diseases remains unknown, an autoimmune aetiology is favoured. However, the dramatic increase in the incidence of FFA over the past decade has caused investigators to also postulate a strong potential environmental aetiology for this disease.^2^

We recommend that patients with FFA/LPP avoid daily use of chemical sunscreens and/or sunscreens containing titanium dioxide. Furthermore. General allergen avoidance is important in controlling scalp symptoms.^9^

In oral squamous cell carcinoma, there is an increased expression of MMPs that play an important role in tumor progression. MMP-2 and MMP-9 play an important role in the cleavage of type IV collagen, facilitating the disruption of the basement membrane and migration of dysplastic cells.^4^

Despite the expression of MMP-2 observed mainly in the lymphocytic band in the lamina propria in oral lichen planus,^4^ it was not noticed in the patients with LPP or FFA in our study.

MMP-1 positively affects the wound-healing process and reduces unwanted scar formation. Therefore, MMP-1 can potentially be used for preventing or treating hypertrophic scars.^10^

We believe that MMPs increase in situations where there is collagen breakdown and disruption of the basal membrane zone, but in cicatricial alopecias, there is mainly accumulation of this substance in the perifollicular zone and fibrosis.

A key limitation of standard immunohistochemistry when studying matrix metalloproteinases (MMPs) is that it detects the presence and location of the protein (total amount, active and inactive), but it does not provide information on its enzymatic activity, which zymography does measure.

MMP-1 and 2 aren’t probably not mediators in the pathogenesis of FFA and LPP.

## ORCID IDs

Adilson da Costa: 0000-0003-0873-6840

Emily Ferreira Salles Pilar: 0000-0003-3417-3586

Julio Cesar Moraes: 0009-0006-4296-6550

Isabel Cristina Gomes Moura: 0000-0002-5549-3426

Moisés Salgado Pedrosa: 0000-0003-0422-9948

## Research data availability

The entire dataset supporting the results of this study was published in this article.

## Financial support

The study received financial support from Fundo de Apoio a Dermatologia (FUNADERM) of the Brazilian Society of Dermatology.

## Authors' contributions

Renata Indelicato Zac: The study concept and design; data collection, or analysis and interpretation of data; statistical analysis; writing of the manuscript or critical review of important intellectual content; data collection, analysis and interpretation; effective participation in the research guidance; intellectual participation in the propaedeutic and/or therapeutic conduct of the studied cases; critical review of the literature; final approval of the final version of the manuscript.

Adilson da Costa: The study concept and design; data collection, or analysis and interpretation of data; statistical analysis; writing of the manuscript or critical review of important intellectual content; data collection, analysis and interpretation; effective participation in the research guidance; intellectual participation in the propaedeutic and/or therapeutic conduct of the studied cases; critical review of the literature; final approval of the final version of the manuscript.

Emily Ferreira Salles Pilar: Data collection, or analysis and interpretation of data; data collection, analysis and interpretation.

Julio Cesar Moraes: Data collection, or analysis and interpretation of data; data collection, analysis and interpretation.

Isabel Cristina Gomes Moura: Statistical analysis.

Moisés Salgado Pedrosa: Data collection, or analysis and interpretation of data; data collection, analysis and interpretation.

## Conflicts of interest

None declared.

## References

[1] Fechine C.O.C., Valente N.Y.S., Romiti R. (2022). Lichen planopilaris and frontal fibrosing alopecia: review and update of diagnostic and therapeutic features. An Bras Dermatol.

[2] Doche I., Romiti R., Hordinsky M.K., Valente N.S. (2020). “Normal-appearing” scalp areas are also affected in lichen planopilaris and frontal fibrosing alopecia: an observational histopathologic study of 40 patients. Exp Dermatol.

[3] Andrade T.C.P.C., Martins T.Y., Oliveira A.M.N., Santiago T.M., Soares C.T., Nakandakari S. (2017). Lichen planopilaris: the importance of early diagnosis. Surg Cosmet Dermatol.

[4] Agarwal N., Carnelio S., Rodrigues G. (2019). Immunohistochemical and clinical significance of matrix metalloproteinase-2 and its inhibitor in oral lichen planus. J Oral Maxillofac Pathol.

[5] Chen B., Cai S., Cui L., Yu T., Qiao K., Su Y. (2025). Novel peptide inhibitor of matrix Metalloproteinases-1 from pufferfish skin collagen hydrolysates and its potential photoprotective activity via the MAPK/AP-1 signaling pathway. J Photochem Photobiol B.

[6] Yip C., Foidart P., Noël A., Sounni N.E. (2019). MT4-MMP: the GPI-anchored membrane- type matrix metalloprotease with multiple functions in diseases. Int J Mol Sci.

[7] Vañó-Galván S., Saceda-Corralo D., Moreno-Arrones Ó.M., Camacho-Martinez F.M. (2018). Updated diagnostic criteria for frontal fibrosing alopecia. J Am Acad Dermatol.

[8] Wong D., Goldberg L.J. (2017). The depth of inflammation in frontal fibrosing alopecia and lichen planopilaris: a potential distinguishing feature. J Am Acad Dermatol.

[9] Ezemma O., Devjani S., Kelley K.J., Senna M.M. (2023). Treatment modalities for lymphocytic and neutrophilic scarring alopecia. J Am Acad Dermatol.

[10] Keskin E.S., Keskin E.R., Öztürk M.B., Çakan D. (2021). The effect of MMP-1 on wound healing and scar formation. Aesthetic Plast Surg.

